# An open‐source tool to visualize potential cone collisions while planning SRS cases

**DOI:** 10.1002/acm2.12998

**Published:** 2020-08-11

**Authors:** Anna Laura Licon, Ara Alexandrian, Daniel Saenz, Pamela Myers, Karl Rasmussen, Sotirios Stathakis, Niko Papanikolaou, Neil Kirby

**Affiliations:** ^1^ Department of Radiation Oncology The University of Texas Health Science Center at San Antonio San Antonio TX USA

**Keywords:** code, cone collision, open source, stereotactic radiosurgery

## Abstract

**Purpose:**

To create an open‐source visualization program that allows one to find potential cone collisions while planning intracranial stereotactic radiosurgery cases.

**Methods:**

Measurements of physical components in the treatment room (gantry, cone, table, localization stereotactic radiation surgery frame, etc.) were incorporated into a script in MATLAB (MathWorks, Natick, MA) that produces three‐dimensional visualizations of the components. A localization frame, used during simulation, fully contains the patient. This frame was used to represent a safety zone for collisions. Simple geometric objects are used to approximate the simulated components. The couch is represented as boxes, the gantry head and cone are represented by cylinders, and the patient safety zone can be represented by either a box or ellipsoid. These objects are translated and rotated based upon the beam geometry and the treatment isocenter to mimic treatment. A simple graphical user interface (GUI) was made in MATLAB (compatible with GNU Octave) to allow users to pass the treatment isocenter location, the initial and terminal gantry angles, the couch angle, and the number of angular points to visualize between the initial and terminal gantry angle.

**Results:**

The GUI provides a fast and simple way to discover collisions in the treatment room before the treatment plan is completed. Twenty patient arcs were used as an end‐to‐end validation of the system. Seventeen of these appeared the same in the software as in the room. Three of the arcs appeared closer in the software than in the room. This is due to the treatment couch having rounded corners, whereas the software visualizes sharp corners.

**Conclusions:**

This simple GUI can be used to find the best orientation of beams for each patient. By finding collisions before a plan is being simulated in the treatment room, a user can save time due to replanning of cases.

## 1 INTRODUCTION

Stereotactic radiation surgery (SRS) is a high‐dose procedure for treating a variety of malignant and nonmalignant diseases. The dosimetric goal for these treatments is to get adequate target coverage while having the dose falloff as sharply as possible outside the target. Linac‐based SRS is a common technique for delivering these treatments. Multiple, non‐coplanar beam arcs are used with this technique to achieve the sharpest falloff possible. Additionally, stereotactic cones can be used for small and/or spherical targets. Compared to multileaf collimators (MLCs), cones produce a more predictable beam output and improved beam penumbra.^1^ However, the cones are closer to the patient than the rest of the linac, which when combined with the non‐coplanar arcs produces potential collisional issues.

A collision with either the patient or treatment couch could result in very serious injuries. This is a possibility that must be checked in person for each arc. Even when checking each arc in person though, there are still two additional issues: the need for replanning a colliding arc and overly conservative arc geometries. Efficiency in a busy clinic is very important. If this collision problem could be addressed before the patient is on the table or before the plan is being physically simulated in the treatment room, there would be great time savings. There are certain table angles that have a very low probability of causing a collision but using only these table angles can produce nonideal dose falloffs.

An open‐source visualization program was developed to help with these issues for intracranial cases. First, this program does not replace in‐person collision evaluation, which is a necessity. It is only meant to avoid replanning and overly conservative beam geometries. The program allows for a visual representation of gantry angles, couch angles and spatial positioning of the gantry, cone, and patient. This program can be used at the time of treatment planning to prescreen a treatment geometry for collisions. This will reduce the probability of replanning and could improve dose falloff for some patients. It is estimated that this visualization ability would help find the optimum beam geometry for at least one third of the cases in our clinic. Additionally, even when employing conservative geometries for problematic target locations, it is estimated that one out of ten cases still needed to be replanned due to a collisional issue.

Some solutions have been used for other treatments and have even taken into consideration the computed tomography (CT) of the patient.^2^, ^3^ Another way has been to create and solve sets of equations to determine collisions based off input angles and couch position.^4^, ^5^ Our solution swiftly models any linac and localizing frame to visualize potential collisions when supplied with beam parameters (e.g., angles, isocenters, etc.). This visual model allows one to see the position of the gantry, cone, and couch throughout the treatment planning phase therefore avoiding workflow issues. Beyond this, instructions are given here for commissioning this software. The program was developed for the MATLAB programming language (MathWorks, Natick, MA), but utilizes code that is fully compatible with GNU Octave to enable easier access.

This method is not any faster or more accurate than those presented in literature.^2^, ^3^, ^4^, ^5^ The value this work brings is through its focus on an open‐source implementation. It takes a significant amount of work to convert mathematical concepts to clinically utilized software. This method was made to be as practical as possible for others to implement. For this reason, the code is included as a supplementary document and this manuscript focuses on the steps needed to put the software into practice. Beyond this, the implementation was made to be as simple and concise as possible so that others can more readily understand and possibly modify the code. More specifically, the fully self‐contained SRS collision code, including comments, is 174 lines long.

## 2 METHODS

### 2.A Overview

Measurements of physical components are incorporated into a script in MATLAB that calculates results based on inputted parameters to produce 3D visuals. To approximate simulated components graphically, all visualized elements are made from a combination of three different three‐dimensional shapes: boxes, cylinders, and ellipsoids. These objects represent the gantry, treatment couch, and a patient safety zone. Although this approximation compromises the resulting quality, it simplifies the geometric model which translates into fast computation times using very low processing power. Computations for this project were calculated on the order of seconds using an i5‐4300U (Intel, Santa Clara, CA) processor. Moreover, by using simple models, it allows for simple conversion of code such that the software can be applied toward other combinations of linear accelerators and components. Visuals are produced by incorporating geometric relationships between the components and their positions relative to machine isocenter. A list of variable descriptions and physical measurements for all the equipment used in the simulations of this study is provided in Table 1.

### 2.B Component visualization

**Fig. 1:**
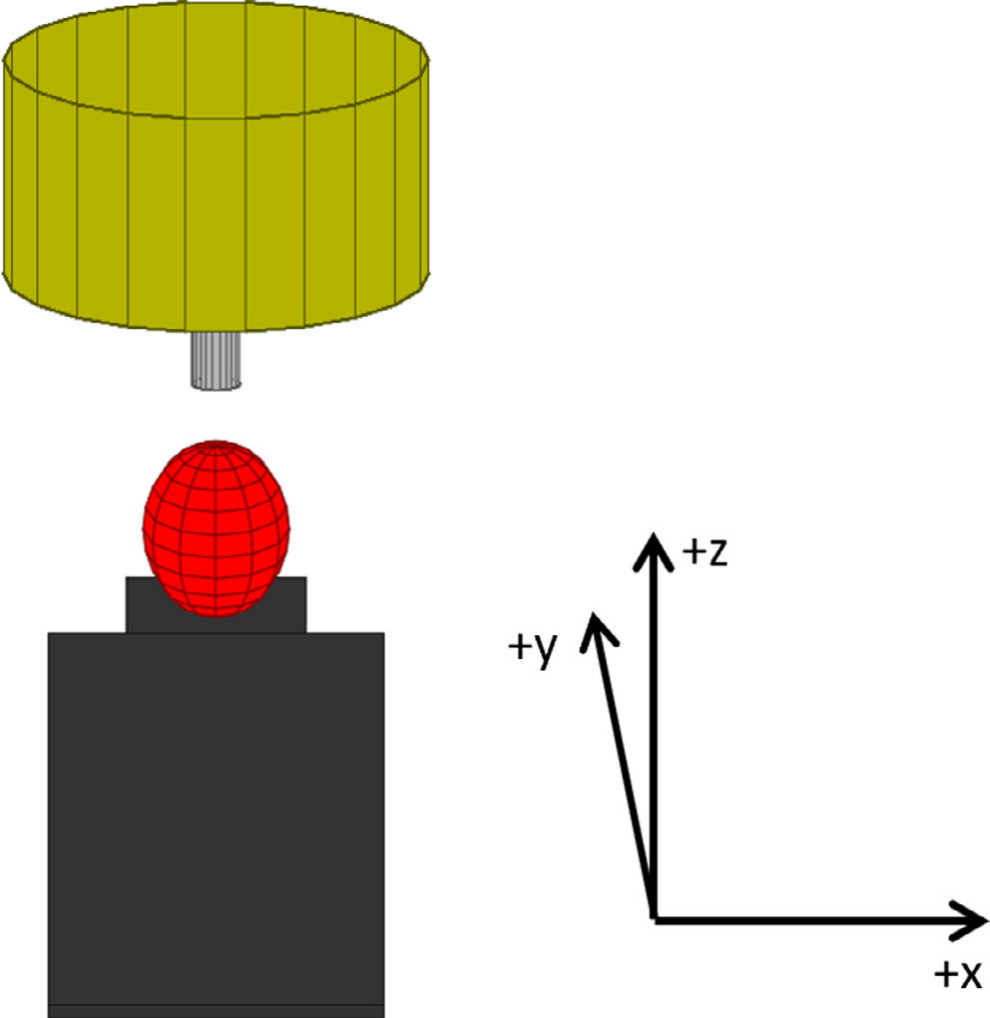
Initial view of the linac, couch, and patient safety zone for the gantry directly overhead, with no couch kick.

Calculations within the software are performed with Cartesian coordinates. More specifically the IEC 61217 fixed system coordinates are utilized. For a head‐first, supine patient, the variable *x* points in the patient left direction, the variable *y* points in the superior direction, and the variable *z* points in the anterior direction (see Fig. 1).

For simplicity, the code relies on the surf function. When given the *x, y*, and *z* coordinates for the vertices of a surface mesh, this code will create a three‐dimensional rendering of it. Beyond this, the function also enables one to modify color and transparency of these surface meshes. Note, however, at the time of writing this manuscript, transparency was not yet supported by GNU Octave.

Creating the representative objects consisted of three steps. *First*, unit objects were defined. These unit objects were a box, a cylinder, and an ellipsoid. The objects were centered on (*x,y,z*) = (0,0,0) and all had a size of 1 in all directions. The unit box had all sides of length equal to 1. For the cylinder, it had a diameter and height equal to 1 and the ellipsoid was a sphere with a diameter of 1. The *second* step was to scale and translate these unit objects to represent the linac, couch, and a patient safety zone for the gantry directly overhead, with no couch kick (see Fig. 1). Boxes are used to represent the head and body of the treatment couch and is one of the options for the patient safety zone. Cylinders visualize the gantry head and cone and the ellipsoid is one of the options for representing the patient safety zone. The *third* step is to perform object rotations. The gantry head and cone are rotated by the gantry angle in the *y* direction. The couch and patient safety zone are rotated by the couch angle in the *z* direction. A mathematical representation of the three steps follows. Let the variable *C* represent a scaling factor, Δ denote a translation, and the variables θ and ϕ represent gantry and couch angle rotations, respectively.

1. Define unit object values*: x_unit_, y_unit_*, and *z_unit_*.
2. Scale and translate. *x_scaled_* = *C_x_⋅ x_unit_* + *Δ_x_; y_scaled_* = *C_y_⋅ y_unit_* + *Δ_y_; z_scaled_* = *C_z_⋅ z_unit_* + *Δ_z_*.
3. Rotate for couch. *x_final_* = *x_scaled_⋅cos(ϕ) − y_scaled_⋅sin(ϕ); y_final_* = *x_scaled_⋅sin(ϕ)* + *y_scaled_⋅cos(ϕ)*. Rotate for gantry. *x_final_* = *x_scaled_⋅cos(θ)* − *z_scaled_⋅sin(θ); z_final_* = *x_scaled_⋅sin(θ)* + *z_scaled_⋅cos(θ)*

After these transformations of the unit objects, the locations can be directly visualized by passing the *x_final_, y_final_, z_final_* coordinates to the surf command.

### 2.C Isocenter, patient safety zone center, and reference point

**Fig. 2:**
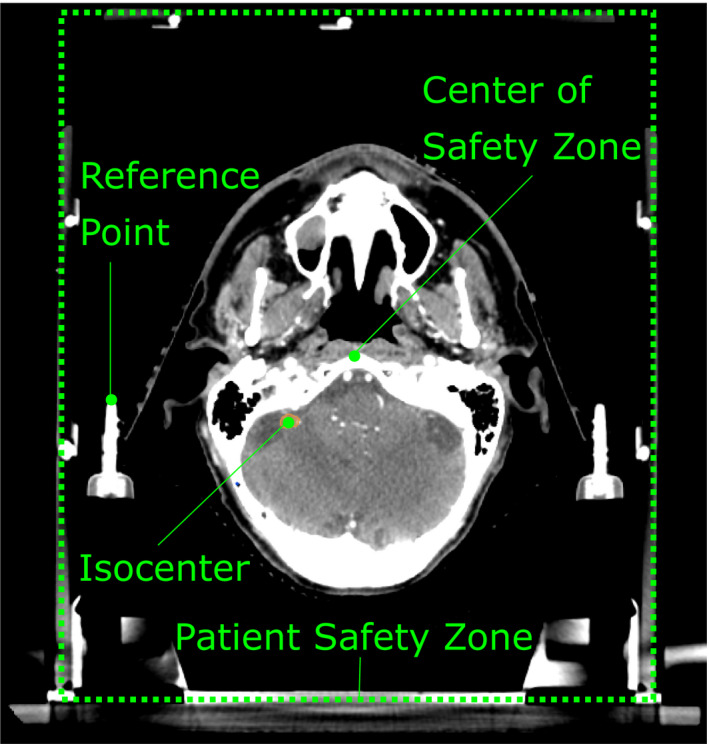
An illustration of the relationship between the isocenter, center of the patient safety zone, and the reference point.

Calculating the object translations for the couch and safety zone relies on knowing the position of three points in space: the treatment isocenter, the center of the patient safety zone, and a reference point. One of the goals of the software is to display the patient safety zone correctly in relation to the treatment (and machine) isocenter. While the treatment isocenter is readily visible in a treatment planning system, the same is not necessarily true for the center of the safety zone. For this reason, an additional reference point, which is visible on the patient CT image is introduced here. During commissioning, the relationship between the reference point and the safety zone is established. Then, during treatment planning, knowing the relationship between the reference point and the isocenter allows one to place the safety zone and treatment couch correctly in relationship to the isocenter. Figure 2 shows an illustration of the three points. In this work, a localization box is used for the patient safety zone and the tip of a rod in the mask base, near the right ear, is utilized for the reference point. Note, not all systems utilize a localization box, but the safety zone concept should be adaptable to other treatment systems.

### 2.D Commissioning

**Fig. 3:**
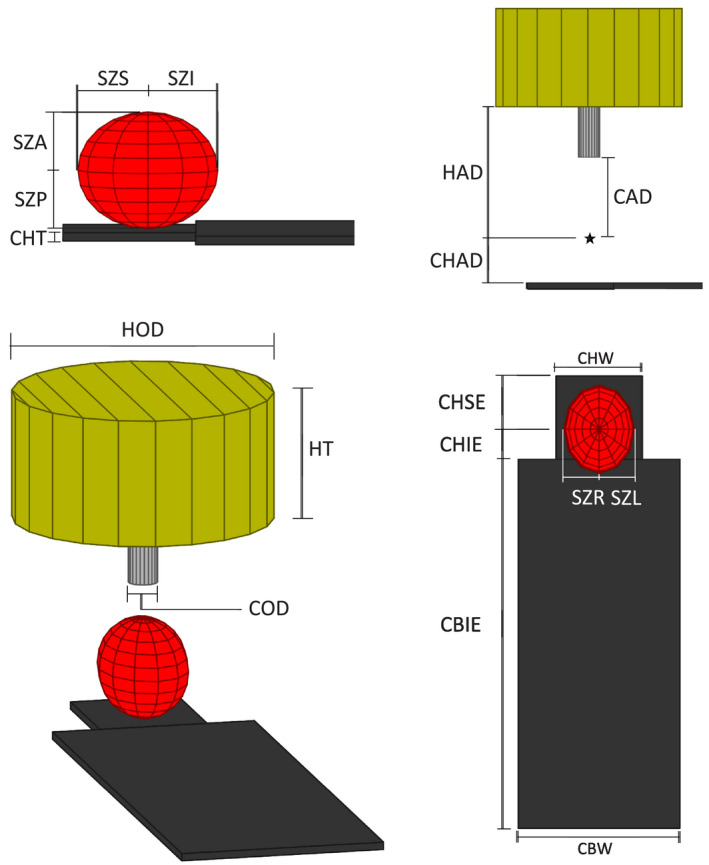
Annotated graphical representation of the measurements needed for the gantry, cone, couch, and patient safety zone (shown as an ellipsoid). The center of this ellipsoid is the center of the safety zone.

Commissioning consists of a set of measurements made to determine the scaling factors, translations, and sign notations for the couch and gantry rotations. Figure 3 provides a physical representation of where each measurement came from along with a description and abbreviation in Table 1. The measurements were taken manually but dimensions from machine blueprints may also be beneficial. These measurements are then manually entered into the MATLAB script. The developed program has no inherent distance units to it. However, it is critical to be consistent in putting measurements in the same units as are used for isocenter shifts. For this manuscript, mm is used for both measurements and isocenter shifts.

The program uses degrees for its angular units. Four data elements are required to characterize the angular conventions of the gantry and couch. The first two are the gantry and couch angle for the setup in Fig. 1. These are referred to here as the gantry initial angle (GIA) and couch initial angle (CIA). The other two are the rotation convention of the gantry and couch. If the gantry angle increases with clockwise rotation, the GICW (gantry increases clockwise) variable is set to 1. Otherwise, it is set to 0. If the couch angle increases with clockwise rotation, the CICW (couch increases clockwise) variable is set to 1. Otherwise, it is set to 0.

The position for the center of the patient safety zone at commissioning is set with the SZLATCOMM, SZAPCOMM, and the SZSICOMM variables (LAT denotes lateral, AP denotes anterior/posterior, and SI denotes superior/inferior). The position for the reference location is set with the REFLATCOMM, REFAPCOMM, and REFSICOMM variables. The locations of these must be found with a measurement tool in the treatment planning system from a CT image. This allows the coordinate conventions to use the same as those that will appear during clinical usage and bypass the need for any additional sign convention definitions. Here, a CT scan was acquired of an empty mask base, with the localization box in place. The center of the localization box was marked with radiopaque markers for visibility. This CT image was imported into Brainlab Iplan and the location of both the center of the safety zone and reference point were found with a measurement tool.

The final information needed to visualize collisions within the software is the sign conventions of the isocenter shifts. A positive shift of the patient safety zone in the x, y, and z directions would move it to the patient left, anterior, and superior. Based on the sign conventions of a treatment planning system, some of these may be reversed. For example, Brainlab conventions for a positive shift in the x, y, and z directions move the patient safety zone to the patient left, posterior, and inferior. For this reason, direction variables (APDIR, LATDIR, and VERTDIR) are used to fix this issue. These variables are made to be either 1 or −1 based on sign conventions. A simple test can be used to find these sign notations, which is entering a large shift (such as 200 mm) in the software and evaluating whether each direction has moved correctly. If it has not, reverse the sign. For Brainlab, APDIR = −1, LATDIR = 1, and VERTDIR = −1.

Beyond the information needed to visualize the collisions, the color of the different elements can also be changed. Red, green, blue (rgb) values can be varied (from 0 to 1) for the safety zone (SZcolor), couch (CHcolor), cone (Ccolor), and gantry head (Hcolor). In this case, these colors were set to red, dark gray, light gray, and dark yellow, respectively. Additionally, the labels presented to the user for the AP, lateral, and sup/inf directions can be changed. For example, in Brainlab, the AP direction is labeled as “Y,” the lateral direction as “X,” and the sup/inf as “Z.” The final step of commissioning is to validate the software and run end‐to‐end tests (see Section 2.F).

### 2.E Software usage

**Fig. 4:**
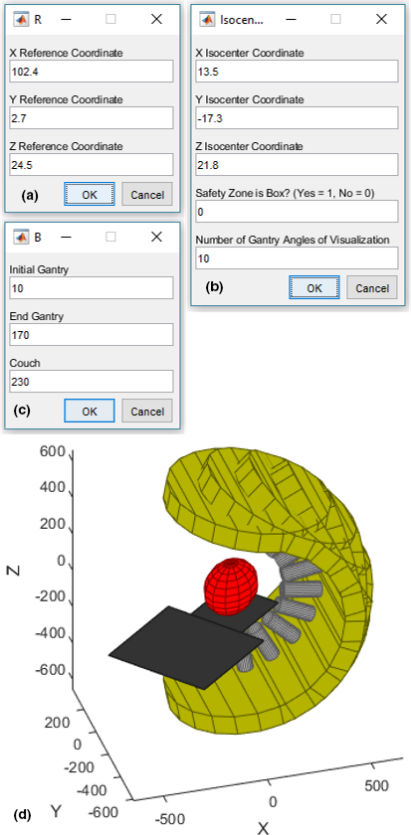
A demonstration of the software usage. (a–c) show the usage in defining the reference location, treatment isocenter, and beam geometry, respectively. (d) shows the corresponding visualization.

**Fig. 5:**
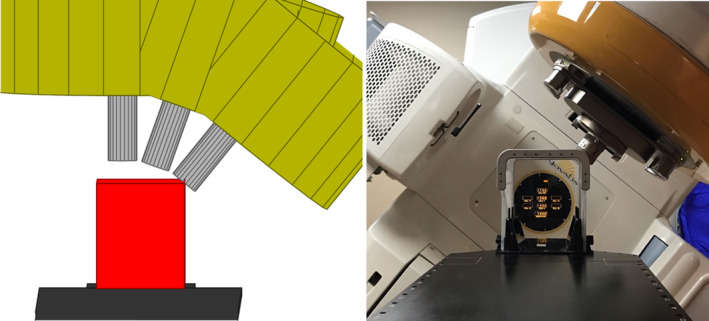
Side‐by‐side demonstration of a collision in the room and on the GUI with the box patient safety zone.

**Fig. 6:**
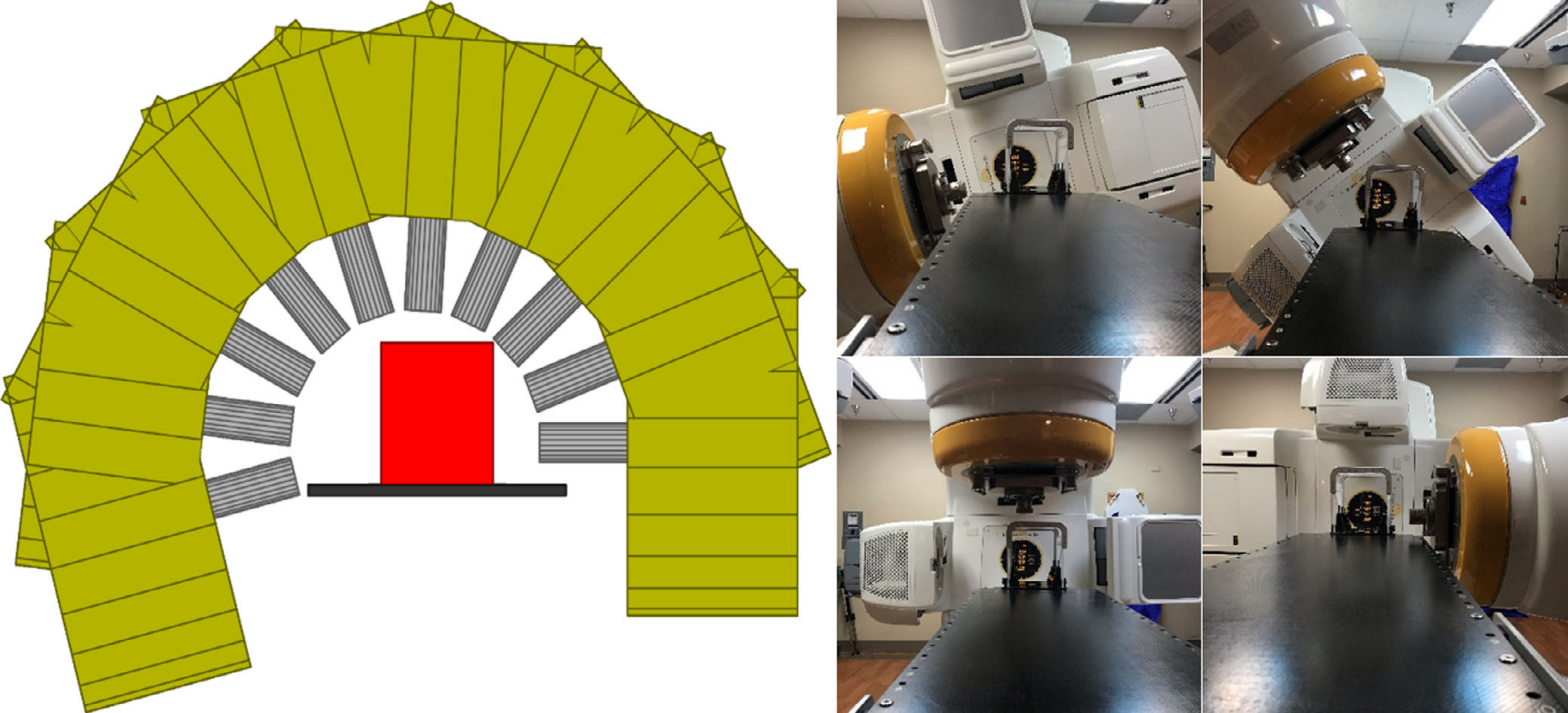
Side‐by‐side demonstration of a noncollision arc in the room and on the GUI with the box patient safety zone.

The software is contained fully within one script. If the folder containing the script is within the defined software path, it can be launched by typing the name of the script (SRS_collision in this case). Otherwise, the script can be opened in either MATLAB or GNU Octave and run directly. When the software is first run, a GUI will open requesting the AP, lateral, and sup/inf coordinates for the reference location in the current plan [see Fig. 4(a)]. Note, this will likely be different than the value established at commissioning. When the wanted values are entered, press the OK button. The next GUI is then launched asking for the analogous locations of the treatment isocenter, whether to represent the safety zone as a box, and the number of gantry angles to visualize between the initial and end angles [see Fig. 4(b)]. Again, press OK to accept the wanted values. The final GUI is then launched with will request the gantry and couch angles to visualize [see Fig. 4(c)]. After selecting OK again, it will show the collision visualization [see Figs. 4(d), 5, and 6]. This image can be rotated to gain access to the best angle for collision analysis. Once the user is finished with this, they close the image, which will launch a configuration menu, where they can input a new beam geometry, isocenter, or reference point and rerun the software. Each of these can be set individually after the initial configuration.

### 2.F Validation and end‐to‐end tests

It is necessary to test the coincidence between the GUI and the treatment room components. A simple way to test this coincidence is to find collision points by translating the couch until there are collisions with each corner while rotating the gantry. Once these collision points are found, the shifts can be replicated in the GUI. This should be repeated with all components: corners of the couch and patient safety zone. Another validation test is to replicate a past clinical patient on both the GUI and the linac and check for coincidence in motion of all parts. This includes ensuring the isocenter shifts are properly replicated for each isocenter tested.

## 3 RESULTS

The measurements needed to create this collision software were gathered in 20 min and easily added to the code. Once the code was altered with the proper dimensions, the end‐to‐end validation was shortly followed. The total time spent on commissioning this, including end‐to‐end validation, was 2 h. Three patients and a total of three isocenters were tested for accuracy between the GUI and the treatment room. Each shift was checked to ensure coincidence with the target coordinates provided by BrainLab. Each treatment arc was visualized in the software and verified in the vault. The arc results were recorded as one of three classifications: (a) the cone will *clear*, (b) the cone is *close* enough (<1 cm) to cause concern for collisions for the patient setup, and (c) the cone *collides*.

Two of the patient plans tested here had collision issues. Tables 2 and 3 show the results for the simulated plans on the software versus the actual in‐room result. The other plan was a plan without collisions which was treated successfully at our clinic. The results for this simulated plan are shown in Table 4. These patient cases included a total of 20 arcs. For the in‐room assessment, two of these arcs were found to collide. The software correctly identified both of these as collisions. Four of the arcs were found to be close with the in‐room assessment. The software also correctly identified all four of these cases. The remaining 14 arcs were found to clear in the room. In the software, 11 of these were also clear, two appeared to be close, and one appeared to collide.

The GUI was tested for coincidence at multiple isocenters, gantry angles, couch kicks, and collimator angles. Figure 5 demonstrates a collision while Fig. 6 demonstrates a noncollision with the GUI replicated next to it. The software and in‐room comparison showed similar results in terms of collisions, close, and complete clears. There were times though when the software appeared closer than the in‐room evaluation. This is due to the treatment couch having rounded corners, whereas the software visualizes sharp corners. For this treatment couch, this distance is 2 cm. For arcs passing by these corners, the cone would appear in the software to be 2 cm closer to the table than in the room.

## 4 DISCUSSION

The GUI provides a fast and simple way to discover collisions in the treatment room before the treatment plan is completed. It is simple to use and can be used to find the best orientation of beams for each patient. By finding collisions before a plan is being simulated in the treatment room, the clinic can save time in the treatment room. This collision software will also provide a better idea of colliding beam geometries to avoid having to make multiple plans.

This GUI was tested in several variations and resulted in clear depictions of the room. The shortcomings of this technique are related to the simple shapes utilized to represent the linac, cone, couch, and patient. More specifically, the sharp corners of the virtual model can over predict collisions near the rounded corners of the couch. Similarly, the patient safety zone utilized here is based on the Brainlab localization box, which is designed to fit over anyone’s head. This would also overpredict cone collisions. In the future, this design could include patient outlines for a more exact design but for now, the simplified geometric shapes provide a conservative approximation for the location of the gantry, couch, and patient.

The GUI was found to be very accurate in replicating the room geometry but did predict collisions in cases that would clear in the room. The only case that did not fit this trend was a collision which resulted from the cone‐locking mechanism. This locking mechanism included a clip which is not a component on the GUI created. In this particular case, the latch passed closer to the table than 1 cm for one arc. The treatment was not replanned though. The couch and gantry angle remained the same, but the collimator was rotated to avoid a collision between the clip on the cone‐locking mechanism and the table.

## 5 CONCLUSION

This simple GUI can be used by the planner, physician or physicist to find the best orientation of beams for each patient. By finding collisions before a plan is being simulated in the treatment room, the clinic can save time due to replanning of cases and avoid overly conservative beam configurations.

## CONFLICT OF INTEREST

No conflict of interest.

6

**Table 1:** Abbreviation and description of each measurement needed for commissioning, along with examples.

Name	Description	Our measurement
SZS	Safety zone distance to superior of isocenter	165 mm
SZI	Safety zone distance to inferior of isocenter	165 mm
SZA	Safety zone distance to the anterior of the isocenter	155 cm
SZP	Safety zone distance to posterior of isocenter	120 mm
SZR	Safety zone distance to the patient right of isocenter	115 mm
SZL	Safety zone distance to the patient left of isocenter	115 mm
COD	Cone outer diameter	750 mm
CHT	Couch head thickness	20 mm
CHAD	Couch head axis distance	120 mm
CAD	Cone axis distance	256 mm
CHW	Couch head width	282 mm
CHSE	Couch head superior extent	200 mm
CHIE	Couch head inferior extent	−113 mm
CBIE	Couch body inferior extent (this can be reduced for visualization purposes)	−2240 mm
CBW	Couch body width	530 mm
HOD	Head outer diameter	670 mm
HT	Head thickness (this can be reduced for visualization purposes)	670 mm
HAD	Head axis distance	437 mm
GIA	Gantry initial angle (in units of degrees)	180
GICW	Gantry increases clockwise (1 = true, 0 = false).	0
CIA	Couch initial angle (in units of degrees)	180
CICW	Couch increases clockwise (1 = true, 0 = false).	1
szlatcomm	Lateral position for the center of the safety zone at commissioning	2
szapcomm	AP position for the center of the safety zone at commissioning	1.8
szsicomm	Sup/inf position for the center of the safety zone at commissioning	0.8
reflatcomm	Lateral position for the reference position at commissioning	103.1
refapcomm	AP position for the reference position at commissioning	4
refSIcomm	Sup/inf position for the reference position at commissioning	23.3
APDIR	Anterior–posterior shift sign convention	−1
LATDIR	Lateral posterior shift sign convention	1
SIDIR	Sup/inf posterior shift sign convention	−1

**Table 2:** Patient #1 had a right orbital gyrus lesion. The original plan used beams 1–4. After plan approval, the beams were simulated on the machine. Beams 3 and 4 were found to collide, thus a new plan was created using beams 5–8.

Beam	Couch angle	Gantry start	Gantry stop	Software (clear, close, collides)	Real (clear, close, collides)
1	180	200	350	Clear	Clear
2	255	160	10	Close	Close
3	205	10	160	Collide	Collide
4	130	200	350	Collide	Collide
5	255	165	45	Close	Close
6	195	55	165	Clear	Clear
7	165	195	315	Close	Clear
8	105	315	195	Close	Clear

**Table 3:** Patient #2 had a left cerebellar lesion. The original plan used beams 1, 2, and 3. After plan approval, the beams were simulated on the machine. Beams 2 and 3 were found to be too close, thus a new plan was created using beams 1, 4, and 5

Beam	Couch angle	Gantry start	Gantry stop	Software (clear, close, collides)	Real (clear, close, collides)
1	180	150	10	Clear	Clear
2	255	10	150	Close	Close
3	105	220	350	Close	Close
4	270	10	140	Clear	Clear
5	225	150	10	Clear	Clear

**Table 4:** Patient #3 had a right cerebellar peduncle lesion. For this treatment, the software was used proactively to find couch angles that were problematic. This showed beams 1 and 2 could be issues. Beams 3 to 7 were chosen to avoid these angles and were used for treatment

Beam	Couch angle	Gantry start	Gantry stop	Software (clear, close, collides)	Real (clear, close, collides)
1	125	190	350	Clear	Clear
2	230	10	170	Collides	Clear
3	180	220	350	Clear	Clear
4	245	80	10	Clear	Clear
5	205	10	90	Clear	Clear
6	145	220	350	Clear	Clear
7	115	350	220	Clear	Clear

## Supporting information


**Data S1**. This is an example SRS cone collision code. It does not replace the need for physical, in‐room cone collision assessment for patients. Those adapting this software for their use must verify the accuracy of these calculations and ensure the safe usage of the code they produce from it.
