# Corrigendum

**DOI:** 10.1002/acm2.13232

**Published:** 2021-03-18

Licon AL, Alexandrian A, Saenz D, Myers P, Rasmussen K, Stathakis S, Papanikolaou N, Kirby N.

An open‐source tool to visualize potential cone collisions while planning SRS cases. *J Appl Clin Med Phys*. 2020;21:40–47.

In the above‐mentioned article, the following acknowledgment section has been included.

Ara Alexandrian was a recipient of a predoctoral fellowship from the Cancer Prevention & Research Institute of Texas (CPRIT)‐funded Research Training Award (RP 170345).

